# Societal costs of sepsis in the Netherlands

**DOI:** 10.1186/s13054-024-04816-3

**Published:** 2024-01-22

**Authors:** Erik C. N. Luijks, Elisabeth C. van der Slikke, Arthur R. H. van Zanten, Jan C. ter Maaten, Maarten J. Postma, Henk B. M. Hilderink, Robert H. Henning, Hjalmar R. Bouma

**Affiliations:** 1 Department of Internal Medicine, University Medical Center Groningen, University of Groningen, P.O. Box 30.001, 9700 RB Groningen, The Netherlands; 2 Department of Clinical Pharmacy and Pharmacology, University Medical Center Groningen, University of Groningen, P.O. Box 30.001, 9700 RB Groningen, The Netherlands; 3 Department of Intensive Care Medicine, Gelderse Vallei Hospital, Ede, The Netherlands; 4 Division of Human Nutrition and Health, Wageningen University Research, Wageningen, The Netherlands; 5 Department of Economics, Econometrics and Finance, Faculty of Economics and Business, University of Groningen, Groningen, The Netherlands; 6 Department of Health Sciences, University Medical Centre Groningen, University of Groningen, Groningen, The Netherlands; 7 National Institute for Public Health and the Environment (RIVM), Bilthoven, The Netherlands

**Keywords:** Sepsis, Economic burden, Costs, Disease burden

## Abstract

**Background:**

Sepsis is a life-threatening syndrome characterized by acute loss of organ function due to infection. Sepsis survivors are at risk for long-term comorbidities, have a reduced Quality of Life (QoL), and are prone to increased long-term mortality. The societal impact of sepsis includes its disease burden and indirect economic costs. However, these societal costs of sepsis are not fully understood. This study assessed sepsis’s disease-related and indirect economic costs in the Netherlands.

**Methods:**

Sepsis prevalence, incidence, sepsis-related mortality, hospitalizations, life expectancy, QoL population norms, QoL reduction after sepsis, and healthcare use post-sepsis were obtained from previous literature and Statistics Netherlands. We used these data to estimate annual Quality-adjusted Life Years (QALYs), productivity loss, and increase in healthcare use post-sepsis. A sensitivity analysis was performed to analyze the burden and indirect economic costs of sepsis under alternative assumptions, resulting in a baseline, low, and high estimated burden. The results are presented as a baseline (low–high burden) estimate.

**Results:**

The annual disease burden of sepsis is approximately 57,304 (24,398–96,244; low–high burden) QALYs. Of this, mortality accounts for 26,898 (23,166–31,577) QALYs, QoL decrease post-sepsis accounts for 30,406 (1232–64,667) QALYs. The indirect economic burden, attributed to lost productivity and increased healthcare expenditure, is estimated at €416.1 (147.1–610.7) million utilizing the friction cost approach and €3.1 (0.4–5.7) billion using the human capital method. Cumulatively, the combined disease and indirect economic burdens range from €3.8 billion (friction method) to €6.5 billion (human capital method) annually within the Netherlands.

**Conclusions:**

Sepsis and its complications pose a substantial disease and indirect economic burden to the Netherlands, with an indirect economic burden due to production loss that is potentially larger than the burden due to coronary heart disease or stroke. Our results emphasize the need for future studies to prevent sepsis, saving downstream costs and decreasing the economic burden.

## Introduction

Sepsis is a potentially life-threatening syndrome of acute organ dysfunction in response to an infection [1]. In 2017, 11 million people died due to sepsis worldwide, which makes sepsis the leading cause of death [2]. In the Netherlands, between 9726 and 20,632 patients with sepsis are admitted to the ICU every year [3]. When including patients with less severe sepsis treated in the general wards, the total sepsis incidence is approximately 58,707 per year [2]. The sepsis-related mortality in the Netherlands is estimated between 8073 and 10,984 cases per year [2–4]. Sepsis survivors are at significant risk for a lower health status and a shorter life span after hospital discharge. As such, sepsis survivors have a lower Quality of Life (QoL), are often in need of physical and medical care, suffer from increased mobility problems, experience cognitive deficits, have lower employment rates, and up to half of the sepsis survivors remain anxious or depressed [5–8]. Next to the significant economic burden of acute mortality due to sepsis, those long-term health effects are expected to have a potentially high economic burden on the Dutch society. Currently, it is unclear what the societal costs of sepsis are.

The total societal costs of a disease consists of its direct economic burden (i.e., medical costs), disease burden, and indirect economic burden. The disease burden can be measured using quality-adjusted life years (QALYs) [9, 10], while the indirect economic burden is formed by productivity loss and increased healthcare use [11]. The medical costs for severe sepsis at the ICU in the Netherlands was estimated at €19,500 per patient in the year 2000 [12], which would amount €27,026 in 2018 after adjusting for economic inflation using the consumer price index [13]. When multiplied with the incidence of sepsis, this would lead to a direct economic burden between €263 and 558 million per year for ICU-treated sepsis patients in the Netherlands [3, 12]. The disease and indirect economic burden of sepsis in the Netherlands are unknown, while insight into the total societal burden is crucial to increase awareness, make informed policy decisions, and reduce sepsis-related costs, among others by initiating a sepsis recovery healthcare programs or funding research to improve early recognition and treatment of sepsis. Therefore, we assessed the disease and indirect economic burden of sepsis in the Netherlands systematically and intelligibly following the guidelines for societal cost–benefit provided by the Dutch Healthcare Institute (in Dutch: Zorginstituut Nederland) [14].

## Methods

### Study design

**Fig. 1:**
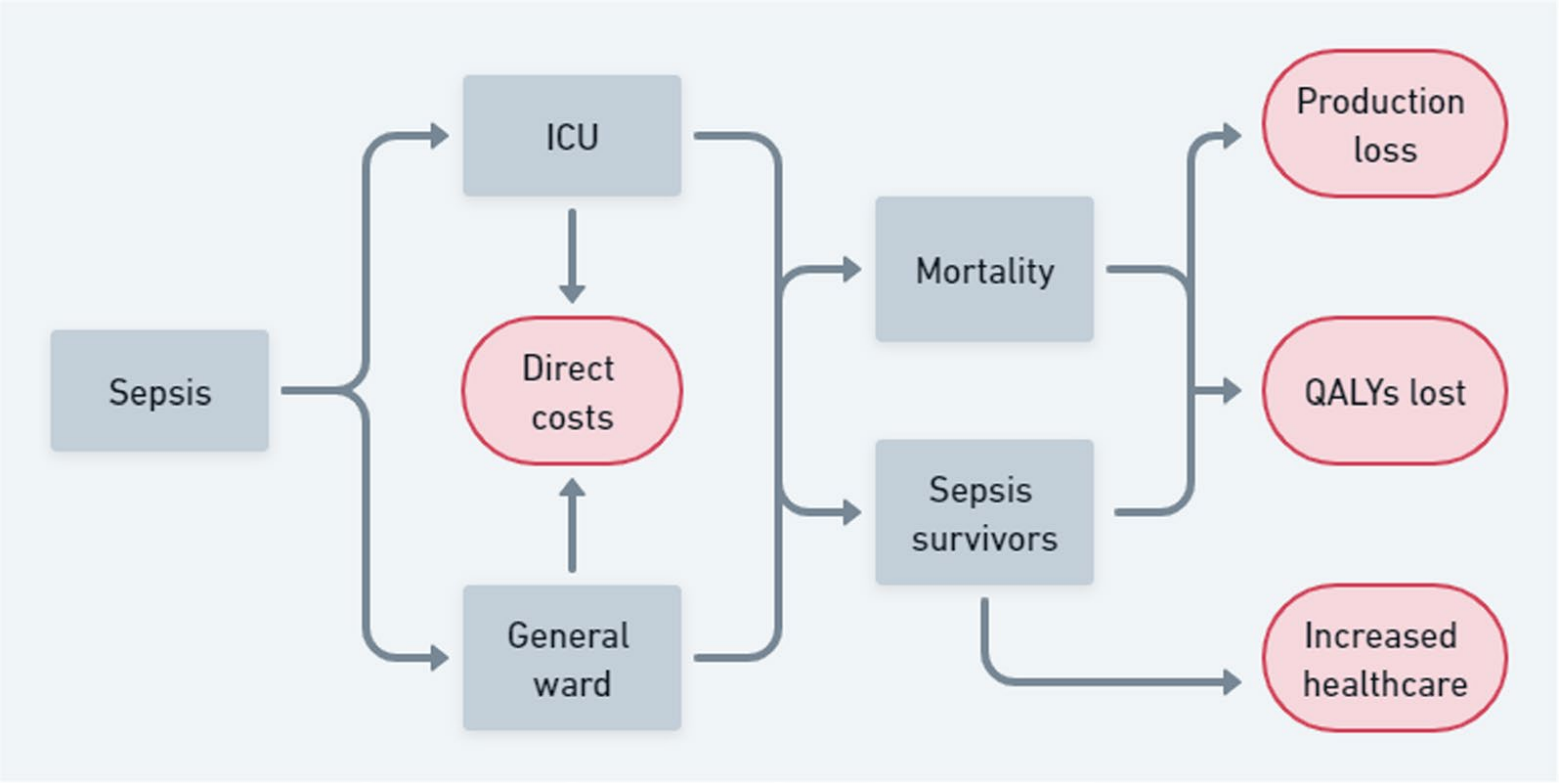
Conceptual model of costs due to sepsis. The burden of sepsis consists of the direct economic costs, indirect economic costs composed of production loss and increased healthcare use after sepsis, and disease burden composed of QALYs. *QALY* quality-adjusted life years, *ICU* intensive care unit

In this study, we estimated sepsis’s disease burden and economic impact. The conceptual model of costs due to sepsis is visualized in Fig. 1. This study followed the guidelines provided by the Dutch Healthcare Institute [14].

### Quality-adjusted life years

Quality-adjusted life years (QALY) is a composite measure of the burden of disease, consisting of premature mortality and the impact of a disease on the quality of life (QoL) [10]. We measured QoL by the EQ-5D, a standardized five-dimension health-related quality of life instrument focusing on mobility, self-care, daily activities, pain/discomfort, and anxiety/depression. First, we assessed the impact of acute sepsis-related mortality on loss of QALYs, using data available for both ICU and ward-treated sepsis patients in the Netherlands [2, 4]. We used Dutch EQ-5D population norms, divided into age categories of five years, to calculate how many QALYs were lost due to mortality caused by sepsis per age group [15]. Next, we assessed the impact of a reduction in health, leading to loss of quality of life (QoL) and QALYs among sepsis survivors. We used EQ-5D data obtained in a Dutch multi-center study comprising 357 sepsis survivors who had been treated at the ICU [16]. However, since EQ-5D data from Dutch sepsis survivors who had not been admitted to the ICU was not available, we used such data from German studies [17–19]. Finally, to validate and generalize our findings, we have repeated our analysis using data from a multi-center study in the UK that comprised 293 ICU-treated sepsis patients [6]. QALYs were calculated with continuous discounting [15], where a discount rate of 1.5% was applied for all future effects and 4% for all future costs in all analyses as recommended by the Dutch Healthcare Institute [14]. The QALYs were expressed in monetary terms according to the value for a QALY advised by the Dutch Institute for Public Health and Environment (in Dutch: RIVM) [20]. All data collected to obtain health and economic burdens are either from 2018 or corrected for inflation using the Netherlands Statistics consumer price inflation index [13].

### Sepsis incidence and mortality

We obtained the incidence rate of sepsis (i.e., ICU and ward-treated patients) in the Netherlands from the Global Burden of Disease study in 2017 [2], while a point-prevalence study performed on 47 Dutch ICUs in 2004 provided the incidence rate of sepsis at the ICU [3]. We derived sepsis-related mortality from the Global Burden of Disease study [2], as well as the point-prevalence study on Dutch ICUs in 2004 [3]. The number of inhabitants in the Netherlands was obtained from the Dutch Statistics Office [13].

### Productivity loss

The indirect economic burden consists of productivity loss and additional healthcare use after sepsis [11]. Productivity loss consists of presenteeism (i.e., working with suboptimal health) and absenteeism (i.e., being absent from work). Productivity loss can be due to three causes: (1) premature mortality, (2) incapacitation to work, and (3) reduced labor productivity [21]. We calculated the productivity loss using two different methods: the friction cost and human capital methods, according to the Dutch guidelines for Economic Evaluations in Healthcare [22], with a friction period of 85 days as recommended by the Dutch healthcare institute [14]. The friction cost estimates societal productivity loss as the short-term costs employers incur in replacing a lost worker [23]. In contrast, in the human capital method, the absence from work due to illness is considered and valued [24]. The productivity loss due to reduced productivity is based on the percentage of annual work-days lost (AWLDs) to the number of working days in the Netherlands. The number of working days per person in the Netherlands was 228 per year, as defined by SEO Economics Amsterdam (in Dutch: Stichting voor Economisch Onderzoek) [25]. The Dutch Institute for Public Health and Environment (in Dutch: RIVM) recommends a formula to calculate AWLDs, accounting for both absenteeism and presentism to work, being AWLDs = QALY * (− 318.0672) + 234.599 [21]. Data about labor costs (i.e., wages, employee benefits, income taxes) and unemployment were obtained from StatLine [13]. Persons between 15 and 65 years old were defined as the working population.

### Healthcare use after sepsis

We obtained the additional healthcare expenditure data after the initial sepsis episode from a study analyzing post-ICU admission healthcare expenditures of sepsis survivors [16]. We corrected the data for inflation in 2017 and 2018, 1.4% and 1.7%, respectively, using the consumer price index available at Statistics Netherlands [13]. We limited the increase in healthcare use to two years post-sepsis because data about increased healthcare use beyond those years was unavailable.

### Statistical analysis

We performed a sensitivity analysis to determine the impact of sepsis on disease burden and indirect economic costs under different sets of assumptions leading to a baseline scenario (i.e., average), low- and high-burden scenario. Definitions of input data and how the values were obtained for the sensitivity analysis are described above, while Table 1 demonstrates the input data of the sensitivity analysis and how the values for the baseline, low- and high-burden scenarios were computed. Data are presented as average (i.e., baseline) cost estimates with results from the sensitivity analysis based on a low-burden scenario and a high-burden scenario between parenthesis.

**Table 1:** Sensitivity analysis

	Baseline scenario		Low-burden scenario	High-burden scenario
Sepsis-related mortality (number per year)	9400		8073	10,984
Total sepsis incidence (number per year)	58,707	46,160	77,794	
Incidence of sepsis at ICU (number per year)	15,179		9726	20,632
Incidence of sepsis at the ward (number per year)	43,528	36,434	57,162	
Duration of QoL decrease post-sepsis (years)	10		5	Life long
Discounting	Continuous discounting; discount rate of 1.5% for all future effects and 4% for all future costs			
Productivity loss after sepsis	20.9%			
QoL decrease after sepsis (EQ-5D)	0.15			
Monthly increase in healthcare expenditure after sepsis	€2,281 (first year); €1,690 (second year)			

Three scenarios were devised to assess the burden and indirect economic costs of sepsis in the Netherlands. The scenarios vary regarding mortality rate, sepsis incidence, and the duration of Quality of Life (QoL) reduction. The baseline input is the average of the highest and lowest sepsis incidence rates obtained from different calculations. Duration of QoL Decrease: Informed by literature and a theoretical model of lifelong reduction

## Results

### Burden of disease

To estimate the disease burden in terms of QALYs lost, we first calculated the disease burden attributable to mortality and subsequently incorporated the impact of a decline in the QoL following sepsis. The Global Burden of Disease study estimates that 58,707 (46,160–77,794; 95% uncertainty interval) patients in the Netherlands are affected by sepsis annually, encompassing both ICU admissions and general ward treatments [2]. A multi-center point-prevalence study indicates that annually, between 9,726 and 20,632 sepsis patients are admitted to the ICU in the Netherlands [3]. First, we estimated the QALYs lost due to sepsis-related mortality by adjusting the in-hospital mortality rate according to the age at death [4]. In 2017, sepsis accounted for 9400 (8073–10,984; low–high burden) deaths in the Dutch population, including both ICU and general ward patients [4]. Hereby, sepsis-related mortality led to 26,898 (23,166–31,577) QALYs lost in 2018, equating to 157 QALYs per 100,000 inhabitants, based on a population of 17,181,084 in the Netherlands [13]. Second, to account for QALYs lost due to diminished QoL among sepsis survivors, we utilized EQ-5D data from a Dutch multi-center study involving 357 individuals who had survived sepsis post-ICU discharge [16]. At 14 months after leaving the ICU, the average EQ-5D index score for these survivors was 0.15 lower than that of the general population [16]. Unfortunately, comparable data for Dutch sepsis survivors who were treated in general wards but not admitted to the ICU are not available. However, a study from Germany has shown that non-ICU-treated sepsis survivors experience similar issues as those treated in the ICU [18]. Together, the reduced QoL among sepsis survivors contributes to an annual loss of 30,406 (1232–64,667) QALYs, or 177 QALYs per 100,000 inhabitants.

**Fig. 2:**
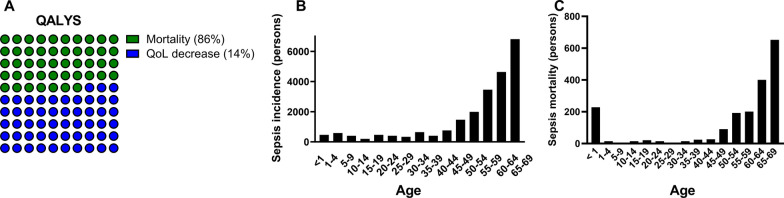
Disease burden, sepsis incidence, and mortality. **A** Burden of disease expressed in QALYs lost due to sepsis, distinguishing between QALYs lost due to mortality (86%) and QALYs lost due to a decrease in QoL among sepsis survivors (14%). **B** Incidence of sepsis across different age groups within the Netherlands. **C** Annual sepsis-related mortality across different age groups within the Netherlands. Subjects exceeding their expected life expectancy at birth were excluded from the figure

To validate and generalize these findings, we repeated our analysis using data from a multi-center study in the UK that included 293 post-ICU sepsis survivors [6], demonstrating a less severe reduction in EQ-5D index score of 0.094. Using this number, the estimated QALY loss would be 14,798 instead of 30,406 lost QALYs. The total number of QALYs lost due to sepsis-related mortality and a reduction in QoL after sepsis was estimated at 57,304 (24,398–96,244) per year, which equals 334 QALYs per 100,000 individuals (Fig. 2). In the Netherlands, this amount of QALY is valued economically at €3.4 (1.5–5.8) billion per year.

### Indirect economic burden

By calculating the indirect economic burden of sepsis, we first assessed the effect of sepsis on production loss, where after we added the effect of additional healthcare use after sepsis. One year after ICU treatment, 47% of previously employed sepsis survivors remain unemployed, and the remaining 53% continue to work, albeit with diminished productivity [26]. Similar effects are observed in sepsis survivors treated in general wards [17, 18]. Initially, the overall drop in productivity post-sepsis was calculated. Based on an average of 228 working days per person annually [25] and a 0.15 decrease in QoL at 14-month post-sepsis [16], productivity decreased by 20.9%. The loss of production in monetary terms was quantified using the average labor cost per age group [13]. Using the friction method, the estimated annual productivity loss from premature mortality was €11.8 (10.2–13.8) million, from inability to work was €71.4 (14.5–95.4) million, and from diminished work productivity was €16.8 (1.6–22.5) million. In total, the friction method demonstrated an annual production loss of €100.0 (18.5–131.7) million, which equals €6 per capita. Conversely, the human capital approach calculated the annual productivity loss due to early mortality at €308.5 (168.3–469.0) million, with an additional loss of €1,996 (116.5–3,810) million due to the inability to work, and around €470.0 (27.5–898.1) million due to reduced work productivity. Altogether, the human capital method estimated the annual production loss at €2,774.5 (312.3–5,177.1) million, which equals €162 per capita. Table 2 presents the results of the sensitivity analyses for both the friction and human capital methods.

**Table 2:** Production loss

	Friction method			Human capital method		
	Baseline scenario	Low scenario	High scenario	Baseline scenario	Low scenario	High scenario
Premature mortality (€ × 10^6^)	11.8	10.2	13.8	308.5	168.3	469.0
Incapacity to work (€ × 10^6^)	71.4	6.7	95.4	1,996.0	116.5	3,810.0
Reduced productivity (€ × 10^6^)	16.8	1.6	22.5	470.0	27.5	898.1
Total production loss (€ × 10^6^)	100.0	18.5	131.7	2,774.5	312.3	5,177.1

This table details the estimated production loss calculated using the human capital method and the friction method. Different scenarios in the sensitivity analysis are presented to illustrate the impact of different assumptions on the estimated production loss

Second, we assessed the impact of sepsis on subsequent healthcare consumption, which could only be computed for those who had survived sepsis following ICU treatment due to the lack of data for non-ICU-treated sepsis survivors. In the Netherlands, the additional healthcare expenses for ICU-treated sepsis survivors were estimated to be €2,281 per month at one year after sepsis and €1,690 at two years post-sepsis in 2016 [16]. After adjusting for inflation, the increased healthcare costs post-sepsis were estimated at €316.1 (128.6–479.5) million.

**Fig. 3:**
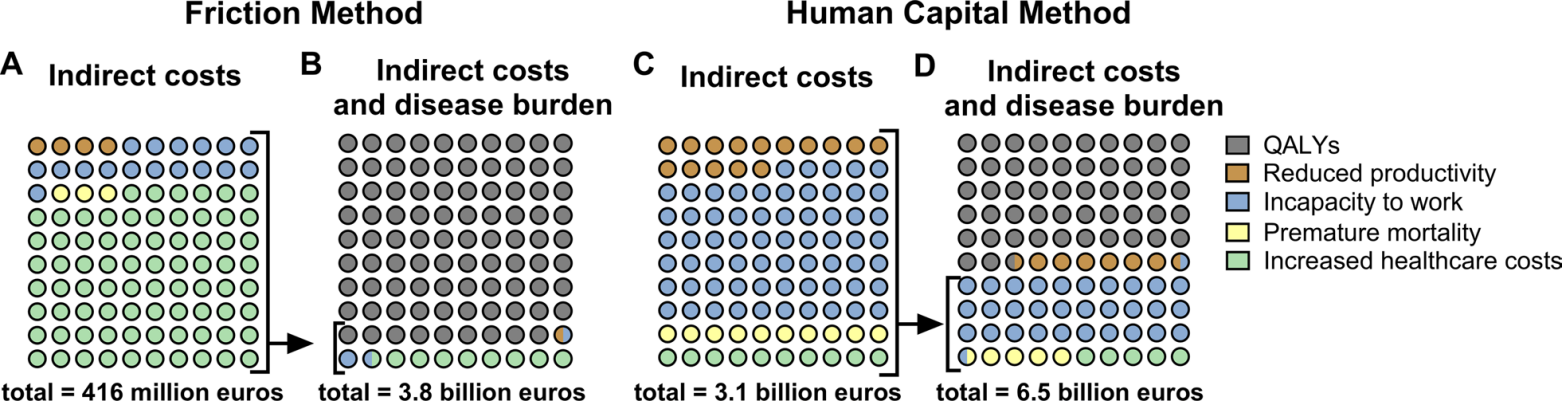
Total indirect economic burden consisting of indirect costs and disease burden of sepsis. **A** Indirect costs of sepsis in the Netherlands using the friction cost approach. **B** Indirect costs and disease burden of sepsis (i.e., loss of QALYs). **C** Indirect costs as assessed through the human capital approach. **D** Indirect costs and disease burden of sepsis (i.e., loss of QALYs)

The total indirect economic burden, comprising both production loss and increased healthcare costs, was calculated using two different methods. Employing the friction method, this burden was estimated at €416.1 (147.1–610.7) million, which equals €24 per capita (Fig. 3A). When accounting for both the indirect economic burden and the loss of QALYs, the total indirect costs escalated to €3.8 (1.6–6.4) billion, equating to €221 per capita (Fig. 3B). In contrast, the human capital method placed the total indirect economic burden at €3.1 (0.4–5.7) billion, which translates to €180 per capita (Fig. 3C). Incorporating both the indirect economic burden and QALYs under this method, the total estimated costs reached €6.5 (2.0–11.5) billion, which equals €378 per capita (Fig. 3D).

## Discussion

### Main findings

Sepsis constitutes a significant health problem in the Netherlands with high mortality and morbidity rates [1–3]. Nevertheless, the total societal burden of sepsis in the Netherlands, encompassing both disease and indirect economic impacts, remains undetermined. Our evaluation places the annual disease burden of sepsis at 57,304 QALYs (valued at €3.4 billion in the Netherlands), with in-hospital mortality accounting for a loss of 26,898 QALYs. The indirect economic burden of sepsis, consisting of production loss and increased healthcare expenditure, is appraised between €416.1 million (€24 per capita; using the friction method) and €3.1 billion (€180 per capita; via the human capital method) annually. Indirect economic burden due to production loss was estimated at €100.0 million by the friction method (€6 per capita) and €2.8 billion according to the human capital method (€162 per capita). Cumulatively, the aggregate cost of the disease burden and indirect economic costs of sepsis ranges from approximately €3.8 billion (€221 per capita; friction method) to €6.5 billion (€378 per capita; human capital method) per year in the Netherlands.

### Interpretation

We are the first to explicitly calculate the disease burden of sepsis in the Netherlands, accounting for 57,307 QALYs that are valued at €3.4 billion each year. In comparison, QALYs lost due to sepsis are approximately two-thirds of the estimated QALYs lost due to COVID-19 in the Netherlands during the pandemic at its peak in 2020 [27]. Further, we demonstrated a profound indirect economic burden due to production loss and increased healthcare expenditure from sepsis in the Netherlands. The indirect economic burden was mainly composed of increased healthcare costs post-sepsis in the friction method and incapacity to work in the human capital method. The indirect economic burden due to production loss was estimated at €100.0 million (€6 per capita) annually using the friction method and €2.8 billion (€162 per capita) annually using the human capital method. In comparison, the indirect economic burden due to production loss calculated by the friction method was €3.8 billion for cancer in 2018 (€524 per capita) [28] and €4.0 billion for diabetes mellitus in 2016 (€540 per capita in 2018) [29]. Thus, according to the friction method the indirect economic costs of sepsis due to lost productivity is ~ 1% of these costs for cancer and diabetes mellitus. However, three quarters of the indirect economic burden of sepsis as assessed by the friction method was due to increased healthcare costs after hospital discharge, which could not be compared with chronic diseases like cancer and diabetes mellitus where healthcare usage comprises diagnosis and treatment of the disease itself, rather than its long-term consequences. The indirect economic burden due to productivity loss assessed by human capital method after cardiovascular disease was estimated at €777 million for coronary heart disease (CHD) and €451 million for stroke in 2021 using human capital method (€42 for CHD and €24 for stroke per capita in 2018) [30]. Hence, lost productivity after sepsis has a fourfold to sevenfold higher indirect economic burden as compared to CHD and stroke, respectively.

The estimated indirect costs due to sepsis align with other European countries such as Austria (€484–686 million; €54–77 per capita) [31], Switzerland (€331–805 million; €46–112 per capita) [32], and Germany (€2,622 million; €32 per capita) [33, 34]. However, comparison across studies remains challenging due to the lack of methodology standardization in cost-of-illness studies. As such, the indirect costs in Austria, Switzerland, and Germany were defined with the human capital method, whereas we used both the friction and human capital method. The friction method results in smaller productivity loss estimates than the human capital method, since the entire period of absence from work due to illness is considered and valued in the human capital method. Additionally, we assumed a lifelong reduced QoL after sepsis for the sensitivity analyses, which have not been performed in previous studies. Based on a notable reduction in QoL that has been observed up to 10 years after sepsis [35, 36] and absence of longer follow-up studies, we assume a lifelong reduction in QoL after sepsis as a plausible scenario. Thus, the estimated indirect economic burden in the Netherlands is considerable and in line with other countries.

The burden of sepsis, as described here for the Netherlands, can likely be generalized to other countries with a similar healthcare system, economy, and comparable demography. To generalize our findings to other countries, we have repeated our analysis using data from the UK. Using data obtained at ICUs in the UK, we estimated a QALY loss of 14,798 due to sepsis-related morbidity as compared to 30,406 using data restricted to the Dutch situation.

### Strengths and limitations

The main strength of this study is the completeness of cost elements, by including productivity loss and healthcare use after sepsis. Furthermore, this study provides a refined model, including sensitivity analysis with two possible scenarios (low and high burden), to estimate sepsis disease and indirect economic burden in the Netherlands. We used both the friction and human capital method to define productivity loss, making comparing our study to different studies easier.

Limitations of this study were the need for CBS Statline, which stratified persons into age categories of 5 years but did not share any data per individual year. The absence of initial EQ-5D assessments in the same group could mean that the observed decline in health status following sepsis might be mistakenly ascribed to preexisting health conditions rather than to sepsis. This could lead to overestimating the quality of life deterioration attributable to sepsis. Furthermore, we did not stratify sepsis into different causes. However, we were interested in sepsis in general. Finally, it is plausible that we have underestimated the total disease burden of sepsis due to a lack of data describing post-sepsis morbidity among non-ICU-treated sepsis patients, although mortality rates were known for this category. Thus, despite these missing data, we were able to include ward-treated sepsis mortality and production losses due to sepsis-related mortality. We were, however, not able to include the QoL decrease, increased healthcare cost and the productivity loss due to absenteeism and presenteeism of sepsis survivors treated at the ward.

### Recommendations

The disease and indirect economic burden after sepsis are high and may be amenable to optimization. Insight into the total societal impact of sepsis is essential to help policymakers make well-advised decisions in the case of funding a sepsis recovery program or funding research into the recognition or treatment of sepsis to decrease costs. We speculate that a comprehensive recovery program for sepsis survivors might be a solution, as it is already seen in ICU survivors, including patients with sepsis [37–40]. In addition, ICU-treated sepsis patients receiving rehabilitation therapy had improved long-term survival rates, without increasing healthcare expenditure [19]. Furthermore, improved recognition of early sepsis might lower disease and indirect economic burden, allowing timely initiation of adequate treatment and preventing the progression to severe sepsis and shock [41]. Together, these interventions could reduce sepsis disease and indirect economic burden.

## Conclusion

Sepsis puts a considerable disease and indirect economic burden on Dutch society, with an indirect economic burden as significant as one-third of the indirect economic burden due to cancer and as significant as the burden due to stroke in the Netherlands. This burden predominantly comprises increased healthcare costs post-sepsis and incapacity to work. Insight into the total disease and indirect economic burden is essential for awareness, informed policy decisions, and cost reduction. The costs are expected to rise due to the aging population and higher life expectancy. These findings indicate that it is relevant to decrease the burden of sepsis, both in terms of cost savings and in reducing the occurrence of long-term complications of sepsis.

## Data Availability

The datasets used and/or analyzed during the current study are available from the corresponding author on reasonable request.

## Abbreviations

AWDLs: Annual work-days lost
ICU: Intensive care unit
QoL: Quality of life
QALYs: Quality-adjusted life years
